# “Engaging, interactive, not boring” – A new innovative tutorial programme for medical students with promising longevity at the University of East Anglia.

**DOI:** 10.1192/j.eurpsy.2024.499

**Published:** 2024-08-27

**Authors:** S. Das, J. Beezhold

**Affiliations:** ^1^Norfolk and Suffolk Foundation Trust, Norwich, United Kingdom

## Abstract

**Introduction:**

Medical students at the University of East Anglia (UEA) complete a psychiatry rotation in the fourth year of their MBBS degree. There are four rotations each academic year, in 2022-2023 there were 24 students per rotation. The rotation consists of two weeks of lectures, a four-week clinical placement, and then a further two weeks of lectures. Students are based across Norfolk and Suffolk for their clinical placements. Although case-based discussions occurred every Wednesday morning via Teams, there were no face-to-face small group teaching sessions during placement.

**Objectives:**

To design an interactive set of tutorials for medical students covering a wide range of psychiatric topics which can be easily delivered by other facilitators.

**Methods:**

Three 1.5 hour tutorials were created: 1)” Psychotic Bingo” - Students have a unique card with terms used in descriptive psychopathology to play Bingo, 2) “Medical ethics, mental health and the law” – Explores the case of Kerrie Wooltorton to discuss the mental capacity act, advanced decisions, and the mental health act, 3) “Team Quiz” – Played in groups and covers the different sub-specialties of psychiatry and pharmacology. Tutorials were only mandatory for students in Norwich (average 11 students per tutorial) due to a large geographical area across placements. Tutorials were delivered for three rotations between December 2022 – May 2023, the initial two rotations by the first author and the third rotation by other facilitators. Facilitators were provided with a tutorial guidance document to ensure consistency. The same feedback form was used to obtain qualitative and quantitative feedback from students at all tutorials.

**Results:**

The table below shows that feedback from students was consistently high, and there was little difference in average students rating between tutorials delivered by the first author and other facilitators. The predominant qualitative feedback was that the tutorials were “very interactive”, “engaging” and “fun”.

**Image:**

**Figure:**
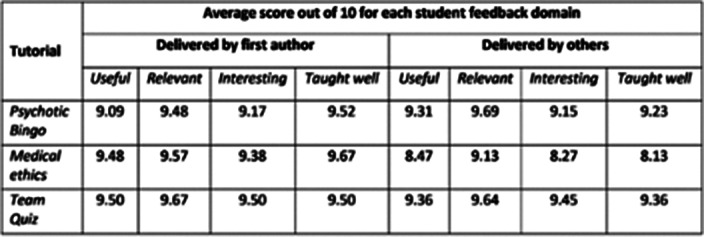

**Conclusions:**

This tutorial programme consistently received excellent feedback. The results show that the tutorials can be effectively delivered by other facilitators whilst maintaining a high standard, which ensures the programmes longevity. The tutorial programme is being formally implemented for all medical students at UEA from October 2023.

**Disclosure of Interest:**

None Declared

